# Efficacy of minimally invasive tubular approaches for management of the lumbar spinal synovial cysts: a meta-analysis

**DOI:** 10.1186/s40001-023-01481-0

**Published:** 2023-11-08

**Authors:** Ying Chen, Pei Yu, Hui Xu, Shenggang Li, Qing Wang, Chunwang Wu, Ji Wang, Fufu Ji, Qiang Huang, Qing Lan

**Affiliations:** Department of Neurosurgery, The Second Affiliated Hospital of Soochow University, Suzhou, Jiangsu 215004 People’s Republic of China

**Keywords:** Lumbar spinal synovial cysts, Traditional percutaneous surgery, Minimally invasive surgery, Tubular retractors, Fusion

## Abstract

The treatment of lumbar spinal synovial cysts (LSCs) which are relatively rare but can cause neurogenic dysfunction and intractable pain has been a controversial topic for many years. Surgical excision of LSCs is the standard treatment for patients in whom conservative treatment options fail. This meta-analysis was undertaken to compare clinical outcomes between minimally invasive approaches using tubular retractors (microscopic vs. endoscopic) and traditional percutaneous approaches for LSCs. Studies reporting surgical management of LSCs were searched in the Cochrane Library, PubMed and Web of Science database. This meta-analysis was reported following the PRISMA Statement, registered in Prospero (CRD42021288992). A total of 1833 patients were included from both the related relevant studies (41 studies, *n* = 1831) and the present series (*n* = 2). Meta-analysis of minimally invasive tubular approaches revealed no statistically significant difference in pain improvement, dural tear, residual cyst, recurrence and operation time between minimal groups with traditional groups (*p* > 0.05). Minimal groups had better Functional improvement of 100% (95% CI 1.00–1.00; *p* < 0.001, *I*^2^ = 75.3%) and less reoperation rates of 0% (95% CI − 0.00–0.00; *p* = 0.007, *I*^2^ = 47.1%). Postoperative length of hospital stay and intraoperative bleeding in minimal groups were also less than traditional groups (*p* < 0.05). Subgroup analysis revealed endoscopic groups had less operation time (*p* = 0.004), and there was no significant difference in the rest. For patients with LSCs but without obvious clinical and imaging evidence of vertebral instability, even when preoperative stable grade 1 spondylolisthesis is present, minimally invasive tubular approaches without fusion may provide the best outcome in surgical management.

**Supplementary Information:**

The online version contains supplementary material available at 10.1186/s40001-023-01481-0.

## Introduction

Synovial cysts often occur in the joints of the limbs (such as the wrist, knee, and ankle) and rarely manifest in the spine [1]. Spinal synovial cysts (SSCs) are asymptomatic, but growth into the spinal canal is an unusual cause of nerve root and/or central canal compression and lead to radiculopathy, intractable back pain, neurogenic claudication, and cauda equina syndrome [2–4]. While synovial cysts have been described throughout the spine, the lumbar spine remains the predominant location [5]. The precise etiology of LSCs remains unclear and the development of LSCs is linked to trauma, spinal instability, and degenerative spondylosis [6, 7]. Current treatment modalities for LSCs include conservation (percutaneous cyst aspiration and steroid injections) and surgery. Surgical approaches include traditional percutaneous approaches (hemilaminectomy or bilateral laminectomy) and minimally invasive tubular approaches (microscopic or endoscopic) [8–10]. Surgical management is indicated following the failure of conservative treatments and can provide significant improvement in clinical symptoms [11]. LSCs treatment’s mainstay is traditional percutaneous approaches. However, they may cause damage to the surrounding muscular, bony and ligamentous structures, potentially increasing segmental instability, particularly in preexisting spondylolisthesis [12]. In recent years, many studies have reported the use of minimally invasive tubular surgery in the treatment of most spinal diseases, but rarely for LSCs [13]. In minimally invasive tubular surgery, the whole synovial cyst is not exposed, which reduces the risk of dural injury [14, 15]. In our study, we successfully excised and cured two patients with LSCs using a microscopic minimally invasive tubular approach. Combining our institutional experience with the meta-analysis results, we proposed minimally invasive excision as an effective treatment for spinal synovial cysts.

## Methods

### Search strategy

**Fig. 1:**
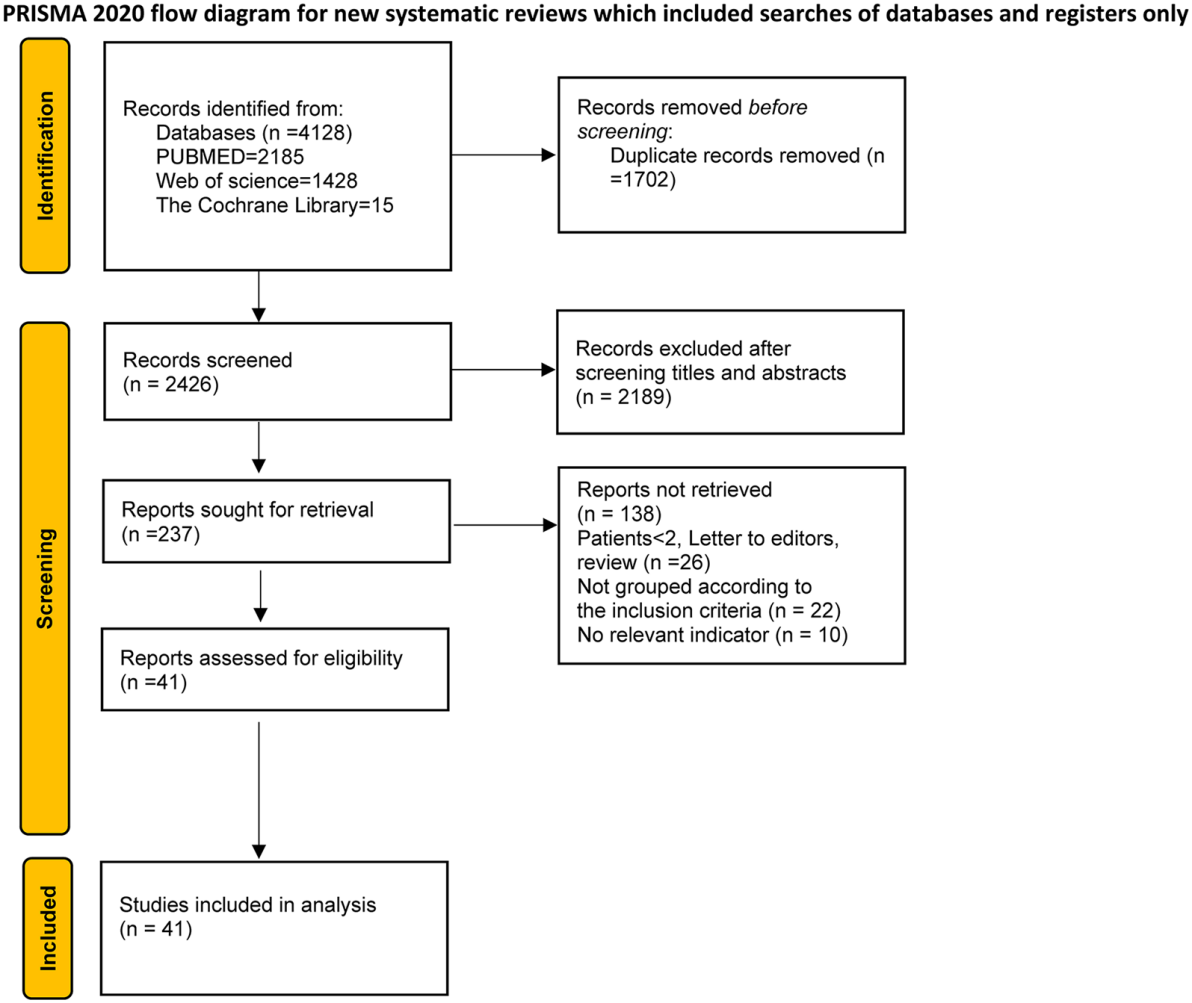
Flowchart of the meta-analysis following the PRISMA

This article was reported following the Preferred Reporting Items for Systematic Reviews and Meta-Analyses (PRISMA) Statement. It was registered at the International Prospective Register of Systematic Reviews (CRD42021288992) [16, 17] (Fig. 1). A comprehensive online search was conducted via Web of Science databases, PubMed and The Cochrane Library on October 17, 2023, using the keywords “spinal synovial cyst”, “spine facet joint cyst”, “paraspinal joint cyst”, “spine degenerative cyst”, “spine ganglion cyst”, “lumbar synovial cysts”. Duplicates and literatures involving synovial cysts of thoracic and cervical levels were excluded. We selected the literature following inclusion and exclusion criteria with no language restrictions. This selection process yielded a total of 41 studies.

### Inclusion criteria (studies were included if they met one of the following criteria)

1. Studies described results of surgical modalities for LSCs.
2. Studies compared the outcome of traditional percutaneous approaches (hemilaminectomy or bilateral laminectomy) with that of minimally invasive tubular approaches.
3. Studies compared the outcome of microscopic with that of endoscopic minimally invasive tubular approaches.
4. All patients included in the study either failed non-surgical treatment or expressed a strong desire for surgery.
5. Intraoperative or histological confirmation of LSCs.
6. All patients included in the study were less than grade 2 spondylolisthesis.
7. The localization of the synovial facet cyst was less than 2 segments of the spine.
8. All patients included in the study were lack of previous spinal surgery in synovial cyst treated segment.

### Exclusion criteria (studies were excluded if they met one of the following criteria)

1. Patients' biometric data (sex, age, preoperative complaints, the operative technique, perioperative complications, follow-up time and outcome) were not provided.
2. Patients with other concomitant conditions that could impair the authors’ ability to determine clinical improvements with surgical treatment of synovial cysts were excluded. For example, a concurrent diagnosis of infection, tumor or metastatic disease, recent spinal fracture, behavioral abnormalities.
3. No distinct operative technique was mentioned.
4. Meta-analysis, systematic review and letters to editors were excluded.
5. Case series and reports with < 2 patients were excluded.

### Data extraction

Two researchers extracted all baseline data and primary outcomes from each qualified study: patient gender, age, relevant medical history, presenting symptoms, imaging findings, surgical management, and follow-up times. Our primary outcome variables were symptom resolution at last follow-up times and cyst recurrences.

### Quality assessment of the selected studies

Two researchers independently assessed each non-randomized study according to Newcastle–Ottawa Quality Assessment Scale (NOS) to assess the risk of bias. The NOS scale evaluated three aspects: cases selection, cases comparability, and exposure ascertainment. Cohort studies were evaluated based on study comparability, patient selection and outcome. Differences will be resolved by consensus or with the help of the senior researcher. Finally, forest plots were charted for pooled results. Publication bias was assessed using Egger's test funnel and plots. When *p* < 0.05, it was statistical significance.

### Statistical analysis

All statistical analyses were performed using Stata 16 (Stata Corp LP) and SPSS statistics 25 (IBM) software. A random-effects model was used to define all pooled outcome measures, and the odds ratio (OR) was estimated with its variance and 95% CI. The prevailing heterogeneity between ORs for the comparable outcomes between different studies was calculated using the *I*^2^ inconsistency test that depicts the percentage of total variation across studies and reflects heterogeneity rather than chance. The *I*^2^ statistics were used for the heterogeneity test. If the *I*^2^ values were < 50%, use the fixed-effects model (FEM) to combine the effect quantity; when *I*^2^ ≥ 50%, the random effect model (REM) was used for meta-analysis [18]. Independent samples test and hypothesis test summary combined the effect quantity.

## Results

A total of 4128 relevant studies were retrieved from PubMed, the Cochrane Library and Web of Science databases. After removing duplicate studies and screening, 41 studies were selected for the meta-analysis (Fig. 1). They included one article in German, two in Spanish and 38 in English. We also included our own two cases. The studies reviewed comprised a total of 1833 patients. The earliest study included in the meta-analysis was published in 2001. Most studies were published in the United States followed by American (Additional file 1: Fig. S1). Traditional percutaneous, microscopic minimally invasive tubular and endoscopic minimally invasive tubular approaches were used in 16, 12 and 10 studies, respectively. Three studies compared outcomes of microscopic minimally invasive tubular approaches with those of traditional percutaneous approaches (Additional file 1: Fig. S2). L4–5 was the most common location of facet cyst (*n* = 1129), followed by L3–4 (*n* = 269) and L5–S1 (*n* = 269) (Fig.S3). Patients had a mean age of 61.7 years and were mostly female (*n* = 978 (53.4%)). The mean follow-up was 35.4 months (range, 2.25–111 months). On radiography, 1208 (94.6%) had radiculopathy, 411 (39.9%) had claudication, and 434 (31%) had preoperative stable grade 1 spondylolisthesis. Only 159 (11.8%) were treated with instrumented fusion. In addition, 1088 (87.1%) patients were experiencing excellent and good outcomes as per Macnab’s criteria or experiencing 0–2 scores as per Nurick at last follow-up. Tables 1 and 2 show disease characteristics included in analyzed studies.

**Table 1:** Case reports and case series reporting patients with cervical degenerative cysts and outcomes

Authors and year	Number of cases	Age (mean ± SD, years)	Intervention	Country	Male:female	Duration of symptoms (mean ± SD, mos)	Location	Radiculopathy (*n*)	Claudication (*n*)	Preoperative spondylolisthesis
Present series	2	80 ± 3	③	China	2:0	1 ± 0.5	L4-5(1); L5–S1(1)	1	0	0
Trummer, M., et al., 2001	19	65 ± 10.4	①	Austria	12: 7		L3–4(2); L4–5(15); L5–S1(2)	19	2	6
Pirotte et al., 2003	46	62.2	①	Belgium	8:38	6.82 ± 3.97	L2–3(1); L3–4(3); L4–5(34); L5–S1(11)	46	11	4
Sandhu, F.A., et al., 2004	17	64.2 ± 10.8	③	American	7: 10		L3-4(1); L4-5(14); L5–S1(2)	17	0	8
Khan, A.M., et al., 2005	39	63.3(43–81)	①	American	28:11		L2–3(3); L3–4(8); L4–5(27); L5–S1(2)	31	34	32
Sehati, N., et al., 2006	19	63.6 ± 9.9	③	American	9:10		L3–4(2); L4–5(16); L5–S1(1)	16	1	2
James, A., et al., 2012	16	66.5 ± 10.7	③	American	6:10		L3–4(5); L4–5(9) L5–S1(2)	9	7	9
Landi et al., 2012	15	66.6	①	Italy	6:9	7.76	L2–3(1); L3–4(1); L4–5(8); L5–S1(5)	15	2	0
Jankowski et al., 2012	11	59		Poland	5:6	2–72	L3–4(1); L4–5(9); L5–S1(1)	9	5	1
Rhee, J., et al., 2012	2	69.5 ± 21.9	③	American	0:2	Many mos	L4–5(2)	2	0	0
Komp, M., et al., 2014	74	52(31–78)	②	Germany	32:62	2.07	L1–2(2); L2–3(4); L3–4(5); L4–5(52); L5–S1(17)	74		
Zhenbo, Z., et al., 2014	24	59.7	①	China	10:14	22.4	L3–4(3); L4–5(15); L5–S1(6)			10
Knafo, S., et al., 2015(1)	18	63.6	①	France	8:12		T12–L1(1); L3–4(1); L4–5(13); L5–S1(5)	15	3	3
Knafo, S., et al., 2015(2)	3	68 ± 12.5	③	France	0:3		L3–4(1); L4–5(2)	3	0	0
Scholz, C., et al., 2015(1)	6	53	①	Germany	1:5	4	L4–5(4); L5–S1(2)			
Scholz, C., et al., 2015(2)	2	55.5 ± 2.1	③	Germany	0:2	1 mos to several years	L4–5(2)	0		
Sukkarieh, H.G., et al., 2015	13	66 ± 11	③	American	5:8		L3–4(4); L4–5(9)	11	2	4
Klessinger, 2016	38	66 ± 10.3		Germany	19:19	6.3	L2-3(1); L3–4(6); L4–5(26); L5–S1(5)	37	1	0
Birch, B.D., et al., 2016	40	65 ± 9.6	③	American	13:27	6.5	L3–4(3); L4–5(29); L5–S1(8)	39	1	15
Denis, D.R., et al., 2016	53	50.5 ± 23.3	③	American	26:27		L3–4(7); L4–540); L5–S1(6)	53		18
Krzok, G., et al., 2016	2	50.5 ± 23.3	②	American	1:1	7.5	L3–4(1); L4–5(1)	2	0	1
Kulkarni et al., 2017	30	67	③	India	18:12		L3–4(4); L4–5(23); L5–S1(3)			8
Bruder, M., et al., 2017	140	65.18	①	Germany	50:90	6.4	L1–2(2); L2–3(3); L3–4(29); L4–5(87); L5–S1(19)	131	42	33
Domenicucci, M., et al., 2017	34	63	①	Italy	13:21	4.73	L2–3(1); L3–4(3); L4–5(20); L5–S1(10)	34		14
Hwang, J.H., et al., 2017	3	64.3 ± 7.5	②	Korea	2:1		L3–4(1); L4–5(2)	3	0	
Lista-Martínez, O., et al., 2017	10	70.2	①	Spain	5:5		L3–4(4); L4–5(6)	8	4	4
Oertel, J.M., et al., 2017	11	59	②	Germany	4:7		L4–5(8); L5–S1(3)	10		4
Vergara, P., et al., 2017(1)	13	70	①	England	7:6		L3–4(1); L4–5(11); L5–S1(1)			4
Vergara, P., et al., 2017(2)	24	68	③	England	13:11		L1-2(1); L3–4(6); L4–5(16); L5–S1(1)			9
Bruder, M., et al., 2018	123	70.8	①	Germany	42:81		L1-2(2); L2-3(2); L3–4(26); L4–5(77); L5–S1(16)			29
Akbary, K., et al., 2019	13	60.3 ± 13	②	Korea	9:4		L3–4(2); L4–5(10); L5–S1(1)	13		17
Heo, D.H., et al., 2019	10	57.3	②	Korea	5:5		L3–4(4); L4–5(6);			
Landriel, F., et al., 2019	19	60.2 ± 11.7	③	Argentina	13:8	6.9	L3–4(2); L4–5(13); L5–S1(3); S1-2(1)	16	2	11
Rahim, T., et al., 2019	283	63.4	①	Germany	97:194	5.5	L1-2(10); L2-3(6); L3–4(59); L4–5(167); L5–S1(41)	283	232	86
Telfeian, A.E., et al., 2019	2	61 ± 14.1	②	American	0:2		L4–5(1); L5–S1(1)	2	0	0
Wu, H.H., et al., 2019	8	62 ± 19.6	②	England	4:4	2.38	L2–3(1); L4–5(5); L5–S1(2)	6	2	0
Rosenstock T., et al., 2020	111	64	①	Germany	55:55	2	L3–4(19); L4–5(67); L5–S1(17); L5–S1(8)	97	42	36
Rolemberg Dantas, F.L.,et al., 2020	50	63.3 ± 9.78	①	Brazil	18:32	5	L3–4(1); L4–5(38); L5–S1(11)	44	10	21
Tacconi, L., et al., 2020	35	52(26–79)	②	Italy	18:17	2.75	L3–4(6); L4–5(28); L5–S1(1)			0
Hellinger, S., et al., 2020	47	60.58 ± 12.32	②	Germany	21:26	3	L3–4 (6); L4–5 (32); L5–S1 (9)	47		0
Page et al., 2021	104	63.2 ± 11.4	①	American	50:54		T12–L1 (1); L1–2 (1); L2-3 (4); L3–4 (13); L4–5 (63); L5–S1 (22)	88	26	
Soriano Sánchez, J.A., et al., 2021	33	62.88 ± 9.92	③	Mexico	8:25	48.76	L3–4(2); L4–5(23); L5–S1(9)	32		
Lalanne, L. B., et al., 2022	69	57.8 (36–79)	①	Chile	26:43	3	L3–4 (16); L4–5 (44); L5–S1 (8); other (1)	69		
Chesney, K., et al., 2022	85	65 (37–86)	③	American	43:42		L2-3 (1); L3–4 (14); L4–5 (61); L5–S1 (10)	81	22	43
Francavilla TL., et al., 2022	117	59 ± 11	③	American	63:54	at least 1.5				23

①: traditional open surgical approach; ②: endoscopic approach; ③: microscope with tubular retractors

**Table 2:** Postoperative outcomes and complications in the included studies

Authors and year	Pre op VAS/ post op VAS (lumbago or leg pain)	Pre op ODI (%)/post op ODI (%)	Recurrence	Reoperation	Residual cyst	Dural tear	Fusion	Operative time (mean ± SD, min)	blood loss (mean ± SD, ml)	Postoperative length of hospital stay (mean ± SD, days)	Follow-up (mean mos)	MacNab (excellent and good)/ Nurick(0–2)
Present series	7/0.5	35.1 ± 6.3/0	0	0	0	0	0	197.5 ± 37.5	35 ± 15	6.5 ± 4.5	13.5	2/2
Trummer, M., et al., 2001			1	1	0	0	0			5	22.7	
Pirotte et al., 2003				4	0	0	0				1–132	
Sandhu, F.A., et al., 2004				1		1	0	97 (50–180)	35 (5–100)	< 1 in 82%	13	16/17
Khan, A.M., et al., 2005			1	4		2	26	231 (92–391)	930 (200–2500)	6.2 ± 3.4	26	
Sehati, N., et al., 2006			0	0	0	2	0	158 (75–270)	31 (10–100)	1 in 68.4%	16	18/19
James, A., et al., 2012	7.6/0.6		0	0		2	0	105 ± 37	< 40		18	14/14
Landi et al., 2012			0	1	0	0					24	
Jankowski et al., 2012			0		0	0	0				12	
Rhee, J., et al., 2012			0	0	0	0	0	74 ± 1.41	27.5	0.6 ± 0.73	12	2/2
Komp, M., et al., 2014	7.6/2.2		2	2		2		22 (14–43)	No measurable		24	71/74
Zhenbo, Z., et al., 2014	7.5 ± 1.7/2.5 ± 0.8		0			0	4	71.7 ± 5	144.1 ± 10.7		58.8	
Knafo, S., et al., 2015(1)			2				2					12/18
Knafo, S., et al., 2015(2)			0				0					2/3
Scholz, C., et al.,2015(1)			0				0				95.5	
Scholz, C., et al., 2015(2)			0				0				36	
Sukkarieh, H. G., et al., 2015	7.8/2.9		0	0	1	0		123 ± 30	44	1.5 ± 0.7	20.8	10/13
Klessinger, 2016			0	1	0	5	0				4.2	29/38
Birch, B.D., et al., 2016			1	2		2		58 ± 18.7	20	0.17 ± 0.07	79	37/40
Denis, D.R., et al., 2016			1	2		2		181.96 ± 3283	42.5	0.28 ± 0.68	14.8	34/40
Krzok, G., et al., 2016				1								
Kulkarni et al., 2017	7.6 ± 0.9/1.6 ± 1.04	61.50 ± 8.4/13.16 ± 5.14	0	0	0	1	0				46.5	30/30
Bruder, M., et al., 2017			8	7	8		0				111	76/81
Domenicucci, M., et al., 2017	7.4/1.3		0	1	0	2	12				28.5	
Hwang, J.H., et al., 2017	6/2	54.83 ± 7.13/27.43 ± 1.27		0		0		55			6	3/3
Lista- Martínez, O., et al., 2017			0	1	0		7				12	6/9
Oertel, J.M, et al., 2017				1		4	0	59 (41–75)			10.5	9/11
Vergara, P., et al., 2017(1)	/4.7		0	1		1		109 ± 37		1.5 ± 0.57	14.9	
Vergara, P., et al., 2017(2)	/0.9		0	0		1		103 ± 27		0.63 ± 0.3	9.4	
Bruder, M., et al., 2018			8	9	8						73.3	108/124
Akbary, K., et al., 2019		65.08 ± 7.95/13.46 ± 5.19			0	0		62.31 ± 14.23	< 50			
Heo, D.H., et al., 2019	7.64 ± 0.71/1.63 ± 1.28	45.35 ± 16.15/15.82 ± 10.21		0	0	0	0	60.1 ± 23.4			10.1	
Landriel, F., et al., 2019	8.3/2.5		0	0		1	5	150.33 ± 6331		2.5 ± 1.78	26	19/19
Rahim, T., et al., 2019						4	5				104.4	240/283
Telfeian, A.E., et al., 2019	7/2	28 ± 2.8/5 ± 1.41	0	0		0					24	2/2
Wu, H.H., et al., 2019	7.75/0.63		0	0	0	0		75.75 + 16.3	Minimal	1.25 ± 0.46	12	7/7
Rosenstock T., et al., 2020			7	23	0	3	16				2.25	
Rolemberg Dantas, F.L.,et a.,2020			0	3	0	3	0			2.18 ± 0.6	87.6	47/50
Tacconi, L., et al., 2020	6.8/2.1		2	4	2	2	0	78 (36–150)			15	27/35
Hellinger, S., et al., 2020	8.07 ± 1.57/1.67 ± 1.32		0	2	0	0	0		Minimal		55.46	37/47
Page et al., 2021				11	0	0					85	77/85
Soriano Sánchez, J.A., et al., 2021	8.24/2.21	41.02 ± 12.58/11.82 ± 10.52	1	3			13	143 (55–360)		2(1–5)	17	272/33
Lalanne, L. B., et al., 2022			1		0	2	69				94.8	63/69
Chesney, K., et al., 2022			1	17	0	1	0	94 (46–183)	22 (5–100)	< 2 in 92%	46	63/81
Francavilla TL., et al., 2022	6.2 ± 2.3/3.1 ± 2.8	46.7 ± 13.5/24.7 ± 19	0	0	0	0	0	72 + 35	52 ± 101	0.085	4.17	

### Pain improvement at the last postoperative follow-up

**Fig. 2:**
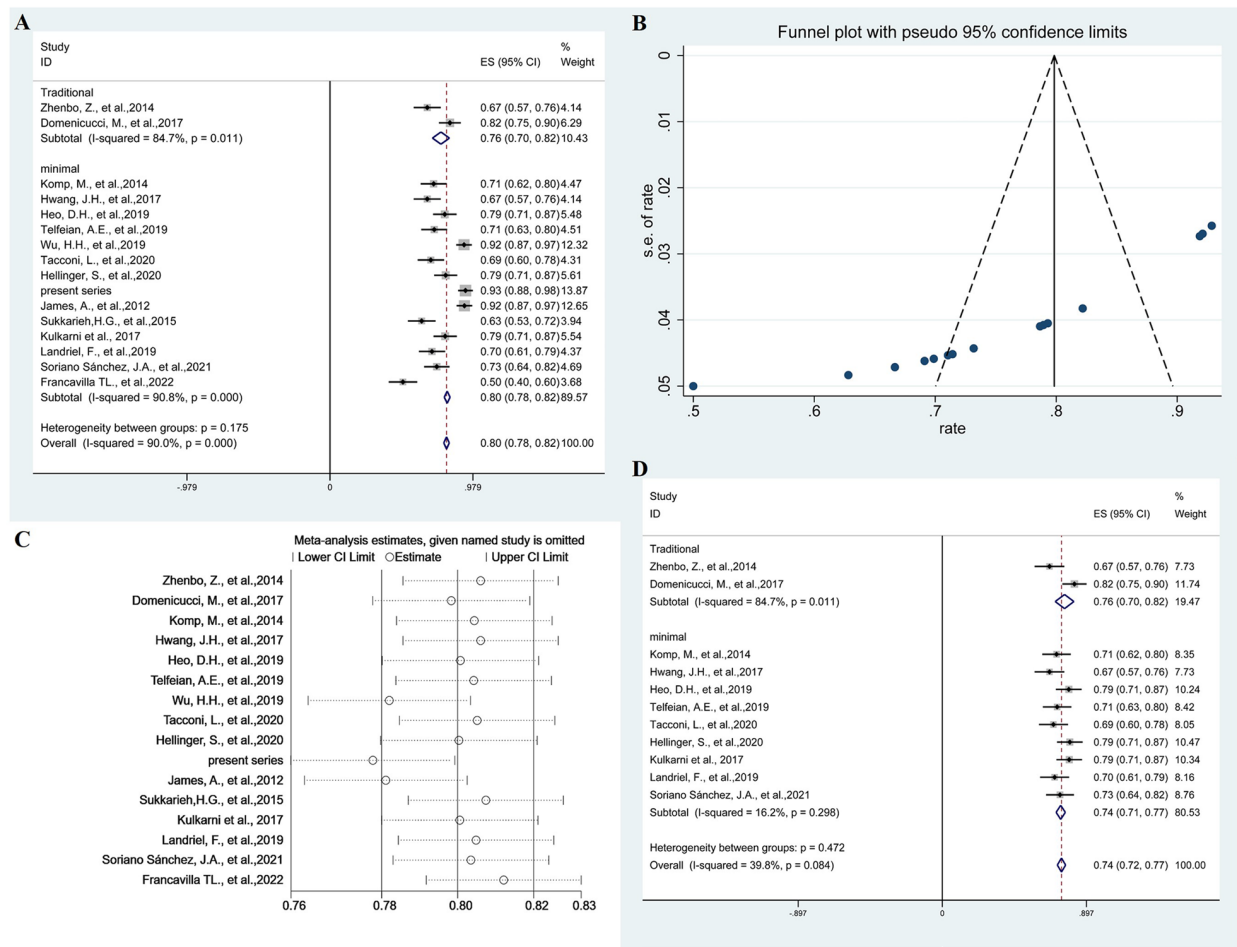
Forest plots showing pooled proportion of percentage change in the VAS scores after surgical resection of cysts (**A**). Funnel plots assessed the publication bias of the change in the percentage of VAS scores after surgical resection of cysts (**B**). Sensitivity analysis using a single-study-removal method (**C**). Forest plots showing pooled proportion of percentage change in the VAS scores after removing studies (**D**)

To facilitate statistical analysis, percentage changes in preoperative and postoperative visual analogue scale (VAS) scores were divided by preoperative VAS scores. This generated a quantitative indicator of pain improvement. The percentage change in VAS scores was 76% (95% CI 0.76–0.82; *p* = 0.011, *I*^2^ = 84.7%, random-effects models) for traditional groups compared with 80% (95% CI 0.78–0.82; *p* < 0.001, *I*^2^ = 90.8%, random-effects models) for minimal groups. This outcome was no significant difference (*p* = 0.175) between traditional groups and minimal groups (Fig. 2A). The Funnel plot were used to assess publication bias of the change in the percentage of VAS scores after surgical removal of cysts, showing publication bias (Fig. 2B). To explore high heterogeneity, we performed sensitivity analysis using a single-study-removal method (Fig. 2C). No changes were seen in terms of the significance of outcome (Fig. 2D). Subgroup analysis of minimal groups showed no significant difference (*p* = 0.204) between endoscopic groups and microscopic groups, which was 79% (95% CI 0.76–0.82; *p* < 0.001, *I*^2^ = 84.8%, random-effects models) and 81% (95% CI 0.79–0.84; *p* < 0.001, *I*^2^ = 94%, random-effects models), respectively (Additional file 1: Fig. S4A). Publication bias was assessed by the funnel plot, suggesting publication bias (Additional file 1: Fig. S4B). When studies were excluded in the sensitivity analysis (Additional file 1: Fig. S4C), no changes were seen in terms of the significance of outcome (Additional file 1: Fig. S4D).

### Functional improvement at the last postoperative follow-up

**Fig. 3:**
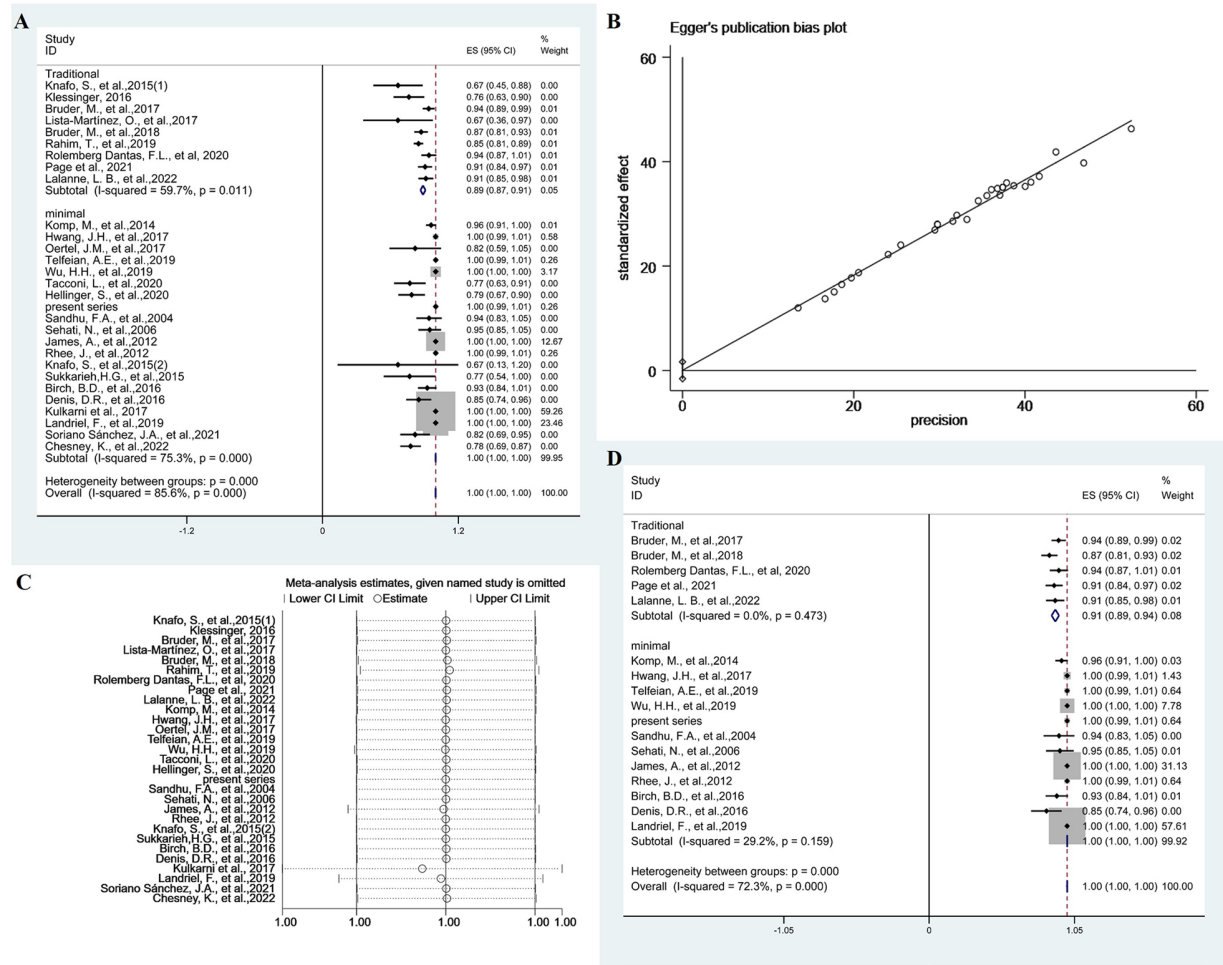
Forest plots showing analysis of studies describing the favorable outcome using Macnab's criteria (excellent and good)/Nurick (0–2) of the last postoperative follow-up to find differences between minimal and traditional groups (**A**). Egger’s test assessing no publication bias (**B**). Sensitivity analysis using a single-study-removal method (**C**). Forest plots showing pooled proportion of percentage change in the favorable outcome after removing studies (**D**)

Analysis of the studies describing favorable last postoperative follow-up outcome using MacNab's criteria (excellent and good)/Nurick (0–2) revealed differences among traditional and minimal groups. The pooled proportion of patients experiencing a favorable outcome following excision of SSCs using traditional groups and minimal groups was 89% (95% CI 0.87–0.91; *p* = 0.011, *I*^2^ = 59.7%, random-effects models) and 100% (95% CI 1.00–1.00; *p* < 0.001, *I*^2^ = 75.3%, random-effects models). This outcome was significantly higher (*p* < 0.001) for minimal groups was than for traditional groups (Fig. 3A). Egger’s regression test yielded a *p*-value of 0.964, suggesting no significant publication bias (Fig. 3B). To explore high heterogeneity, we performed sensitivity analysis using a single-study-removal method (Fig. 3C). No changes were seen in terms of the significance of outcome (Fig. 3D). Subgroup analysis of endoscopic groups and microscopic groups with tubular retractors was 100% (95% CI 1.00–1.00; *p* < 0.001, *I*^2^ = 79.0%, random-effects models) and 100% (95% CI 1.00–1.00; *p* < 0.001, *I*^2^ = 75.1%, random-effects models), respectively. The difference between the two groups was not statistically significant (*p* = 0.811) (Additional file 1: Fig. S5A). Egger’s regression test yielded a *p*-value of 0.207, suggesting no significant publication bias (Additional file 1: Fig. S5B). To explore high heterogeneity, we performed sensitivity analysis using a single-study-removal method (Additional file 1: Fig. S5C). No changes were seen in terms of the significance of outcome (Additional file 1: Fig. S5D).

### Dural tear

**Fig. 4:**
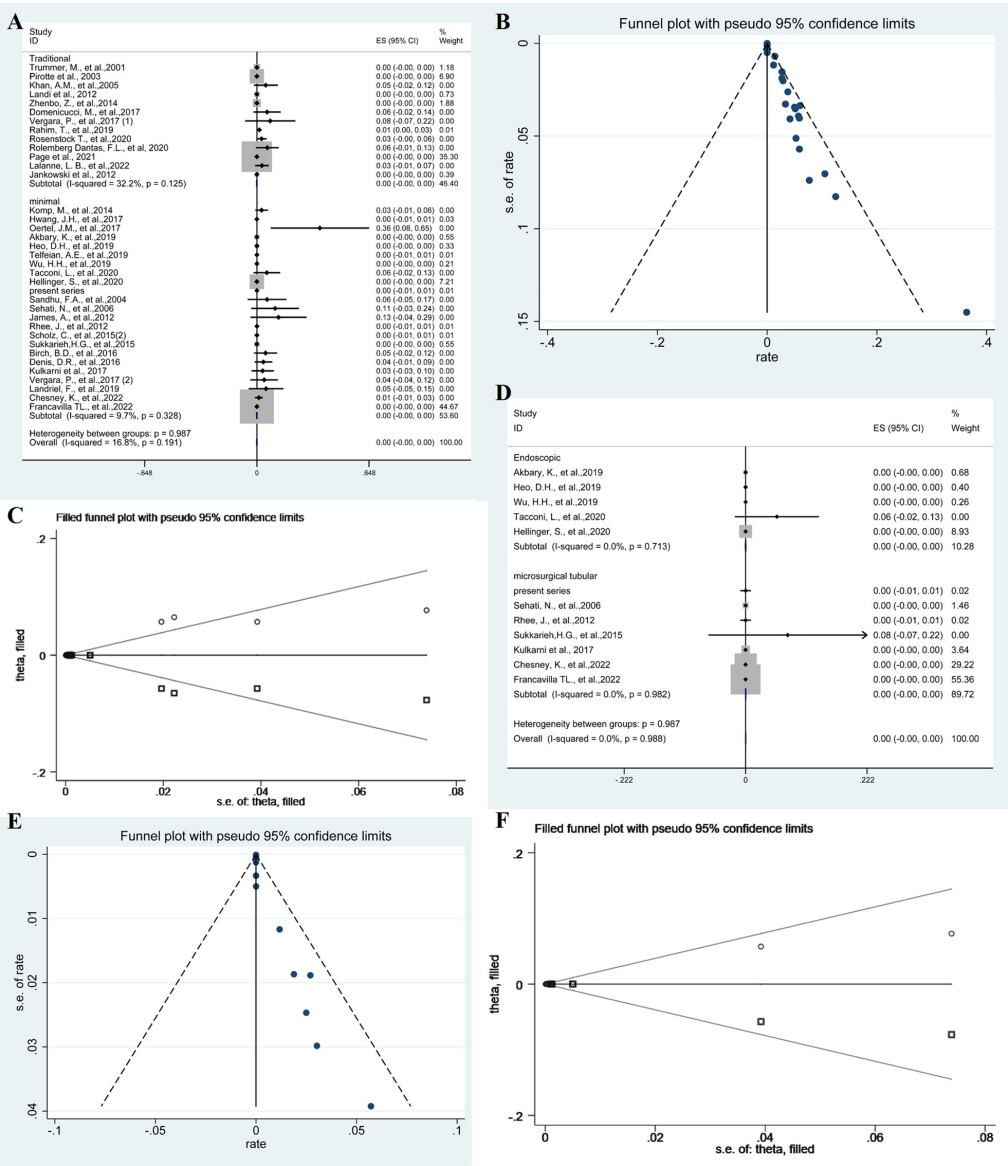
The forest plot showing the pooled proportion of dural tears. Studies were homogeneous with an *I*^2^ value < 50% (**A**). Funnel plots assessed the publication bias of the pooled proportion of dural tears (**B**). The trim and filling method showing the pooled proportion of dural tears (**C**). Forest plots showing pooled proportion of dural tears after minimal resection of cysts (**D**). Funnel plots assessed the publication bias of the pooled proportion of dural tears after minimal resection of cysts (**E**). The trim and filling method showing the pooled proportion of dural tears after minimal resection of cysts (**F**)

According to the meta-analysis results, the pooled proportion of dural tears in traditional and minimal groups was 0% (95% CI − 0.00–0.00; *p* = 0.125, *I*^2^ = 32.2%, fixed-effects models) and 0% (95% CI − 0.00–0.00; *p* = 0.191, *I*^2^ = 16.8%, fixed-effects models). The difference in dural tear between the two groups was not significant (*p* = 0.987) (Fig. 4A). The Funnel plots were used to assess publication bias of dural tears, suggesting publication bias (Fig. 4B). To explore publication bias, we performed trim and filling method. After adding 18 studies, the results were still not statistically significant (*p* = 0.976) and did not reverse (Fig. 4C). Subgroup analysis of endoscopic groups and microscopic groups with tubular retractors was 0% (95% CI − 0.00–0.00; *p* = 0.125, *I*^2^ = 32.2%, I2 = 79.0%, fixed-effects models) and 0% (95% CI − 0.00–0.00; *p* = 0.306, *I*^2^ = 13.7%, I2 = 23.5%, fixed-effects models), respectively. The difference between the two groups was not statistically significant (*p* = 0.981) (Fig. 4D). The Funnel plots were used to assess publication bias of dural tears after minimal resection of cysts, suggesting publication bias (Fig. 4E). To explore publication bias, we performed trim and filling method. After adding 12 studies, the results were still not statistically significant (*p* = 0.986) and did not reverse (Fig. 4F).

### Residual cyst

**Fig. 5:**
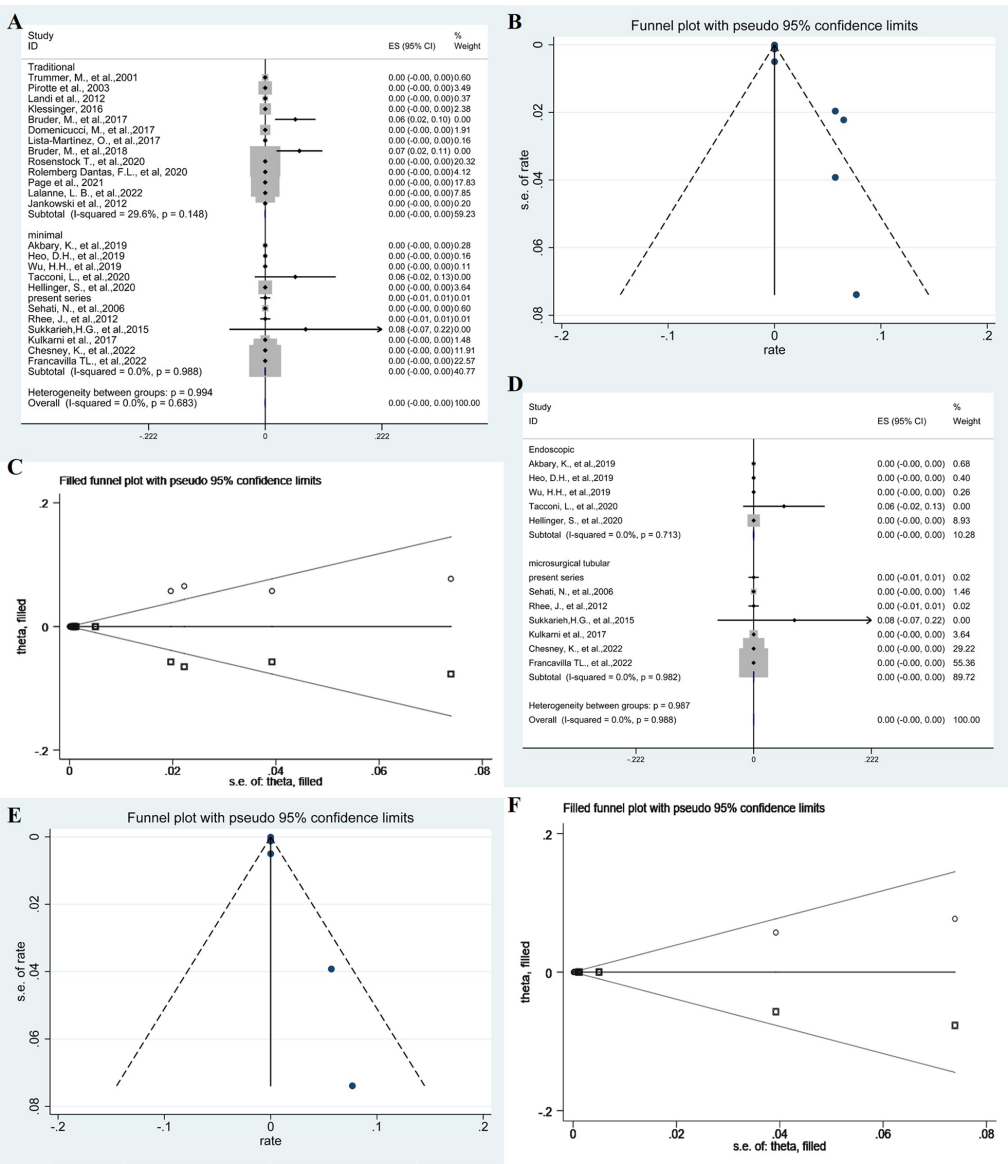
The forest plot showing the pooled proportion of residual cyst. Studies were homogeneous with an *I*^2^ value < 50% (**A**). Funnel plots assessed the publication bias of the pooled proportion of residual cyst (**B**). The trim and filling method showing the pooled proportion of residual cyst (**C**). Forest plots showing pooled proportion of residual cyst after minimal resection of cysts (**D**). Funnel plots assessed the publication bias of the pooled proportion of residual cyst after minimal resection of cysts (**E**). The trim and filling method showing the pooled proportion of residual cyst after minimal resection of cysts (**F**)

The meta-analysis revealed a pooled proportion of residual cyst of 0% (95% CI − 0.00–0.00; *p* = 0.148, *I*^2^ = 29.6%, fixed-effects models) and 0% (95% CI − 0.00–0.00; *p* = 0.988, *I*^2^ = 0%, fixed-effects models) in traditional and minimal groups, respectively. The difference in residual cyst between two groups was not significant (*p* = 0.994) (Fig. 5A). The Funnel plots were used to assess publication bias of residual cyst, suggesting publication bias (Fig. 5B). To explore publication bias, we performed trim and filling method. After adding 12 studies, the results were still not statistically significant (*p* = 0.978) and did not reverse (Fig. 5C). Subgroup analysis of endoscopic groups and microscopic groups with tubular retractors was 0% (95% CI − 0.00–0.00; *p* = 0.713, *I*^2^ = 0%, fixed-effects models) and 0% (95% CI − 0.00–0.00; *p* = 0.982, *I*^2^ = 0%, fixed-effects models), respectively. The difference between the two groups was not statistically significant (*p* = 0.988) (Fig. 5D). The Funnel plots were used to assess publication bias of residual cyst after minimal resection of cysts, suggesting publication bias (Fig. 5E). To explore publication bias, we performed trim and filling method. After adding 6 studies, the results were still not statistically significant (*p* = 0.984) and did not reverse (Fig. 5F).

### Recurrence

**Fig. 6:**
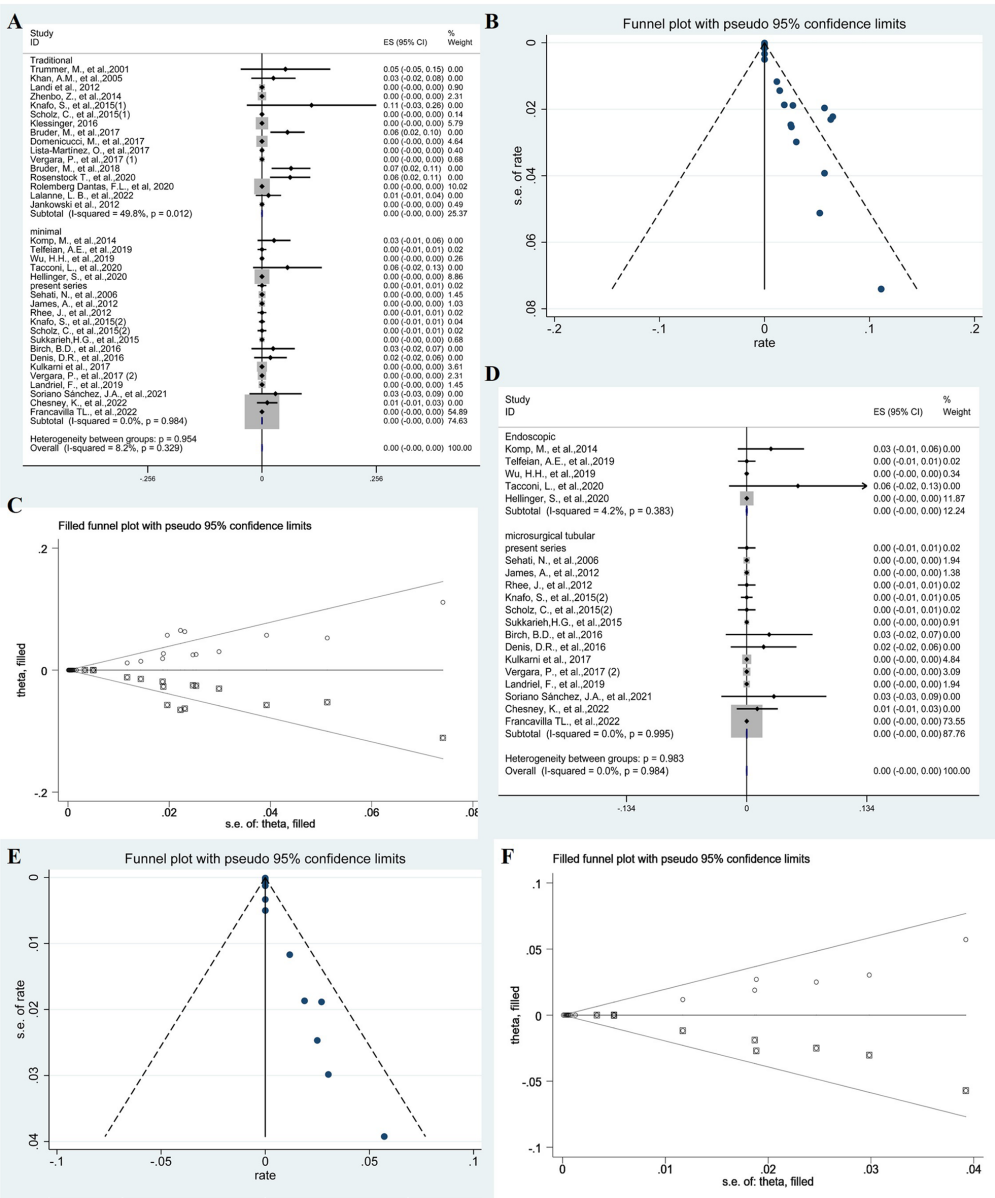
The forest plot showing the pooled proportion of recurrence. Studies were homogeneous with an *I*^2^ value < 50% (**A**). Funnel plots assessed the publication bias of the pooled proportion of recurrence (**B**). The trim and filling method showing the pooled proportion of recurrence (**C**). Forest plots showing pooled proportion of recurrence after minimal resection of cysts (**D**). Funnel plots assessed the publication bias of the pooled proportion of recurrence after minimal resection of cysts (**E**). The trim and filling method showing the pooled proportion of recurrence after minimal resection of cysts (**F**)

The meta-analysis revealed a pooled proportion of recurrence of 0.07% (95% CI 0.05–0.09; *p* = 0.050, *I*^2^ = 42.9%, fixed-effects models) and 0% (95% CI − 0.00–0.00; *p* = 0.984, *I*^2^ = 0%, fixed-effects models) in traditional and minimal groups, respectively. The comparison of subgroup between two groups was not significant (*p* = 0.954) (Fig. 6A). The Funnel plots were used to assess publication bias of recurrence, suggesting publication bias (Fig. 6B). To explore publication bias, we performed trim and filling method. After adding 18 studies, the results were still not statistically significant (*p* = 0.978) and did not reverse (Fig. 6C). Subgroup analysis of endoscopic groups and microscopic groups with tubular retractors was 0% (95% CI − 0.00–0.00; *p* = 0.383, *I*^2^ = 4.2%, fixed-effects models) and 0% (95% CI − 0.00–0.00; *p* = 0.995, *I*^2^ = 0%, fixed-effects models), respectively. The difference between the two groups was not statistically significant (*p* = 0.983) (Fig. 6D). The Funnel plots were used to assess publication bias of recurrence after minimal resection of cysts, suggesting publication bias (Fig. 6E). To explore publication bias, we performed trim and filling method. After adding 11 studies, the results were still not statistically significant (*p* = 0.984) and did not reverse (Fig. 6F).

### Reoperation

**Fig. 7:**
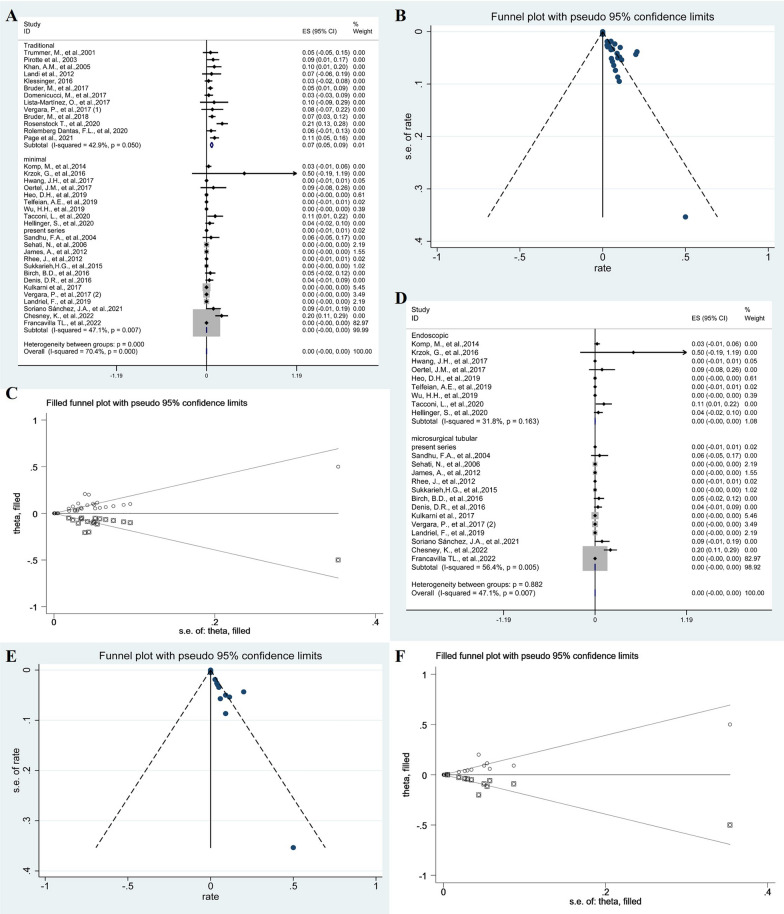
The forest plot showing the pooled proportion of reoperation. Studies were homogeneous with an *I*^2^ value < 50% (**A**). Funnel plots assessed the publication bias of the pooled proportion of reoperation (**B**). The trim and filling method showing the pooled proportion of reoperation (**C**). Forest plots showing pooled proportion of reoperation after minimal resection of cysts (**D**). Funnel plots assessed the publication bias of the pooled proportion of reoperation after minimal resection of cysts (**E**). The trim and filling method showing the pooled proportion of reoperation after minimal resection of cysts (**F**)

The meta-analysis revealed a pooled proportion of reoperation of 6% (95% CI 0.02–0.10; *p* = 0.697, I2 = 0%, fixed-effects models) and 0% (95% CI − 0.00–0.00; *p* = 0.007, *I*^2^ = 47.1%, fixed-effects models) in traditional and minimal groups, respectively. The comparison of subgroup between two groups was significantly significant (*p* < 0.001) (Fig. 7A). The Funnel plots were used to assess publication bias of reoperation, suggesting publication bias (Fig. 7B). To explore publication bias, we performed trim and filling method. After adding 18 studies, the results were still not statistically significant (*p* < 0.001) and did not reverse (Fig. 7C). Subgroup analysis of endoscopic groups and microscopic groups with tubular retractors was 0% (95% CI − 0.00–0.00;* p* = 0.163, *I*^2^ = 31.8%, random-effects models) and 0% (95% CI − 0.00–0.00; *p* = 0.005, *I*^2^ = 56.4%, random-effects models), respectively. The difference between the two groups was not statistically significant (*p* = 0.983) (Fig. 7D). The Funnel plots were used to assess publication bias of reoperation after minimal resection of cysts, suggesting publication bias (Fig. 7E). To explore publication bias, we performed trim and filling method. After adding 11 studies, the results were still not statistically significant (*p* = 0.988) and did not reverse (Fig. 7F).

### Blood loss, operation time and postoperative length of hospital stay

**Fig. 8:**
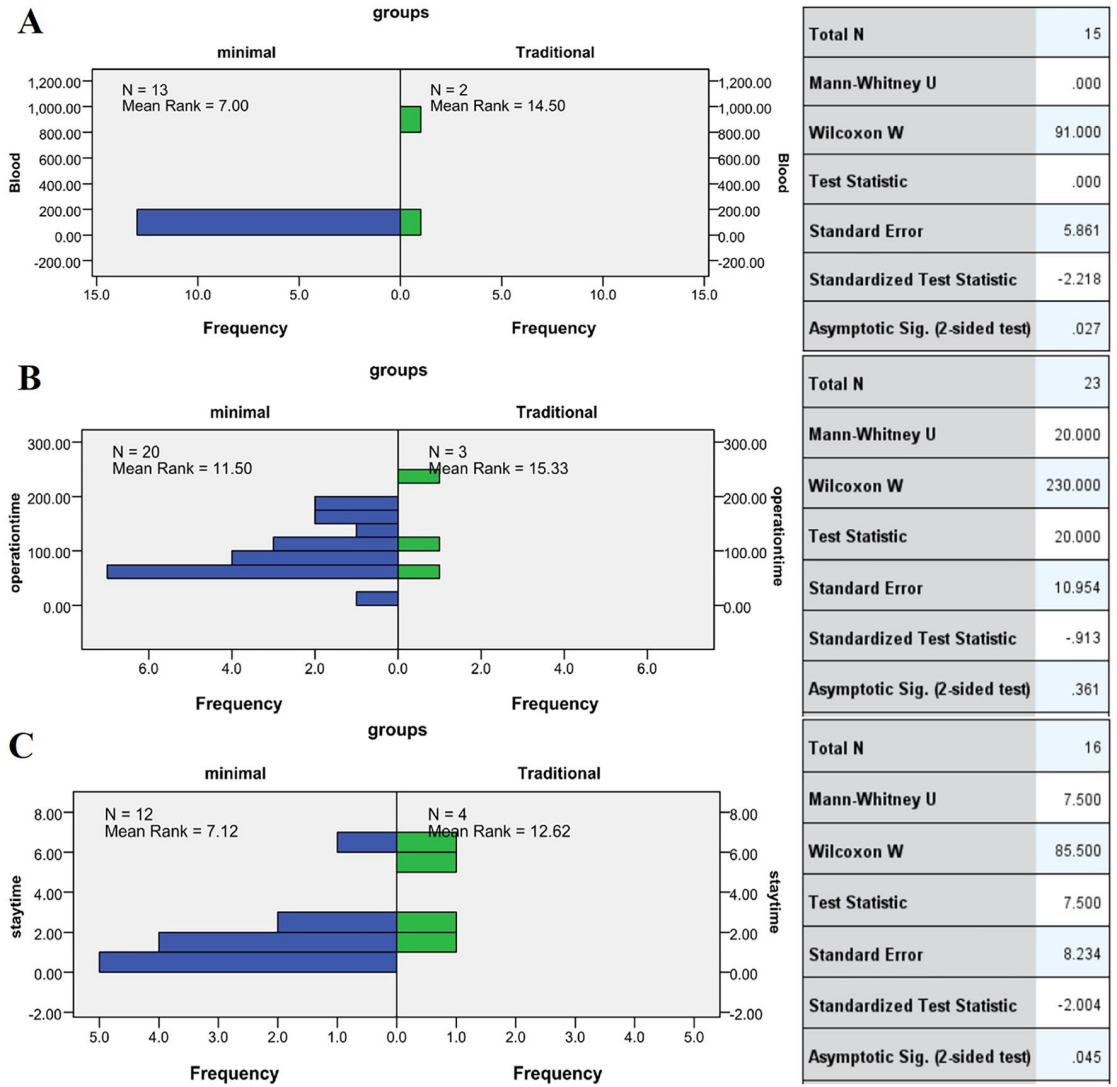
Independent samples test the blood loss between traditional and minimal groups (**A**). Independent samples test the operation time between traditional and minimal groups (**B**). Hypothesis test summary about the postoperative length of hospital stays between traditional and minimal groups (**C**)

For surgical characteristics, independent samples test or hypothesis test summary showed a significant difference in blood loss (*p* = 0.027) (Fig. 8B), operation time (*p* = 0.361) (Fig. 8B) and postoperative length of hospital stay (*p* = 0.045) (Fig. 8A) between minimal and traditional groups. However, the difference in blood loss (*p* = 0.395) (Additional file 1: Fig. S6A), operation time (*p* = 0.004) (Additional file 1: Fig. S6B) and postoperative length of hospital stay (*p* = 0.833) (Additional file 1: Fig. S6C) between the microscopic with tubular retractors and endoscopic groups was not statistically significant.

## Discussion

LSCs are relatively rare but can cause significant symptoms including symptomatic radiculopathy and neurogenic dysfunction. Surgery with the goal of cyst excision or rupture is decompress the affected nerve root. Now, surgeries may be shifting away from larger, more invasive surgeries in favor of minimally invasive options. More minimally invasive surgeries have become a routine procedure for the management of various spine pathologies, e.g., disk herniation, stenosis and schwannoma compression. To the best of our knowledge, this is the most up to-date systematic review and meta-analysis on outcomes and complications of LSCs treatment. We estimated overall outcomes and complications for each surgical approach.

### Outcome

In this review article, we pooled data from all the studies describing the results of surgical resection of LSCs to identify safer and more effective surgical management of LSCs. The pooled proportion of favorable outcomes and the percentage change were higher in the minimal groups were than in traditional groups. It is worth emphasizing that we detected publication bias by the Egger’s test on all findings and used trim and filling method to test the stability of our results. Besides, our analysis showed a significant difference between minimally and traditional groups in postoperative length of hospital stay and blood loss. We also found that minimal approaches minimized incision length, soft tissue trauma, blood loss and disruption of ligamentous and bony structures. In addition, they could produce a better clinical outcome, including complete excision of the cyst, decreased postoperative pain and reduced hospitalization period, and were more cost-effective than traditional surgery. Interestingly, we found that the probability of reoperation in the minimal groups was lower than that in the traditional groups. Moreover, minimally invasive microscopic approaches with tubular retractors are considered far more challenging than conventional approaches due to a high risk of the residual cyst, dural tear and recurrence [19–21]. However, our analysis revealed no significant difference in risk of dural tear, residual cyst, recurrence, and reoperation between minimal and traditional groups. Compared to microscopic excision of LSCs, endoscopic groups with tubular have less operative time. Other analyses revealed no statistically significant difference between subgroups. However, we should also consider operative time (with the associated learning curve involved for surgeons) and accessibility to theater and equipment to perform this specialized surgery [22–25]. In order to better understand this technology, we have made a tabulated summary of the key findings and also comparative literature of the endoscopic approach to other lesions (Additional file 1: Table S1) [25–28].

### Complications

Recent studies reviewed described spontaneous resolution after cyst rupture, triggering a local inflammatory reaction because of the presence of prostaglandins, proteases, and cytokines [29–32]. Because of this phenomenon, cysts strongly adhere to the dura mater due to intermittent small ruptures, hindering cyst resection and increasing the risk of incidental durotomy. Most scholars believe that the traditional surgical approach is the safest approach for excision of synovial cysts, whether decompression or fusion [11, 27]. It seems reasonable to consider that the microscopic with the tubular approach is far more challenging than the conventional one because of risk of epidural hematoma, durotomy and cerebrospinal fluid leak, especially in narrower epidural spaces [12]. However, many studies have reported safe removal of spinal canal synovial cysts using minimally invasive microscopic approaches with tubular retractors and endoscopic approaches. Similarly, many studies have reported using endoscopic approaches and minimally invasive microscopic approaches with tubular retractors in the treatment of LSCs, demonstrating that LSCs can be safely resected with good outcomes [33–35]. In our study, of the 38 patients with incidental dural tears during minimally invasive tubular approach, 88% were managed conservatively or through primary repair.

### Recurrence

In the meta-analysis, joint destabilization was one of the causes of recurrent intraspinal synovial cyst, specifically spondylolisthesis. The presence of spondylolisthesis varied between 23 and 88% (mean, 31.5%) [36]. In our study, we obtained a similar result. Of the 1601 patients, 31% had preoperative spondylolisthesis, supporting, fusion as a first therapeutic choice in most cases [37]. Using minimally invasive surgery, Rolemberg, Scholz and Denis all found no difference in recurrence of radiculopathy, back pain and cyst between patients with decompression alone and those with decompression and fusion [4, 37, 38]. Gupta et al. considered that fusion surgery prolongs surgery and, overall, has more surgery-related risks than sole decompression of SSCs. It also presents additional problems including screw loosening, adjacent level degeneration or breakage [39]. In our meta-analysis, in traditional groups, 31% of patients had preoperative spondylolisthesis, whereas 15.7% underwent fusion. In minimal groups, 27.24% of patients had preoperative spondylolisthesis, whereas 4.02% underwent fusion. Lastly, in the endoscopic groups, 18.64% of patients had preoperative spondylolisthesis, and none underwent fusion. There was no difference in recurrence among the three groups and minimal groups had better functional improvement and less reoperation rates. The analysis results also proved that decompression alone was enough to achieve good results in preoperative stable grade 1 spondylolisthesis patients and low incidence of secondary fusion surgery. Our analysis revealed no difference in postoperative functional recovery, cyst recurrence and reoperation between decompression alone group and decompression with fusion group showed (*p* > 0.05). This conclusion is inconsistent with Khan et al. [40]. A possible explanation for this discrepancy is that their study probably compared only the curative effect difference between fusion and without fusion surgery and ignored the impact of surgical methods [41–43]. Minimally invasive surgery preserves small joints, reduces damage to the surrounding muscles, bones and ligaments, and prevents iatrogenic segmental instability to the greatest extent, especially in preexisting spondylolisthesis [44, 45].

Of the two cases we included in our study, case 1 was a patient with lung metastases from colorectal cancer and case 2 was a patient with chronic myeloid leukemia. Although the two patients had longer intraoperative time due to underlying comorbidities, they recovered well after surgery. Case 2 was hospitalized for a long time due to comorbid fever and high inflammatory indexes after operation. However, on the first day after the operation, he could go to the ground and preoperative low back pain and radiculopathy had been relieved. In our experience, minimally invasive surgery for spinal synovial cysts is characterized by less intraoperative bleeding and damage to the surrounding muscles, bones and ligaments, hence, a better option for patients with severe underlying diseases.

## Limitations

Here, we present the most extensive and first meta-analysis of cases of LSCs. We analyzed patient data collected for nearly 20 years, explicitly compared surgical outcomes and characteristics in patients treated with different surgical approaches and objectively evaluated them from multiple angles. However, our study had some limitations. First, our literature search yielded only three studies study directly comparing the differences in LSCs resection between the microscopic approach through tubular retractors and the traditional surgical approach. Second, as in all meta-analysis studies, our patient populations were subject to heterogeneity in surgical outcomes and patient characteristics. We primarily minimized this bias during initial screening process by excluding studies that reported differing presenting symptoms, comorbidities, and disease etiologies, which were the primary causes of heterogeneities. Third, there are currently no any prospective studies about spinal synovial cysts. A prospective randomized study may provide further insights into the optimal treatment of LSCs. Randomized prospective studies could also provide evidence for safer and most effective surgical management of LSCs.

## Conclusion

Based on patient-specific anatomy of spinal and synovial cysts, we recommend minimally invasive surgical techniques for patients with LSCs. In addition, for patients without obvious clinical and imaging evidence of vertebral instability but with a preoperative stable grade 1 spondylolisthesis, minimally invasive surgery without fusion is adequate primary surgical treatment due to the overall good clinical outcome and low incidence of reoperation, without overtreatment.

### Supplementary Information

**Additional file 1: Fig. S1.** The bar chart shows the annual distribution of published studies and the countries of origin of the studies. **Fig. S2.** The bar chart shows the annual distribution of published studies and the surgical methods used in the studies. **Fig. S3.** Graph showing LSCs locations (T12–L1 n=2; L1–2 n=18; L2–3 n=27; L3–4 n=269; L4–5 n=1129; L5–S1 n=269; S1–2 n=10). **Fig S4.** Forest plots showing pooled proportion of percentage change in the VAS scores after minimal resection of cysts (A). Funnel plots assessed the publication bias of the change in the percentage of VAS scores after minimal resection of cysts (B). Sensitivity analysis using a single-study-removal method (C). Forest plots showing pooled proportion of percentage change in the VAS scores after removing studies (D). **Fig. S5.** Forest plots showing subgroup analysis of minimal groups described the favorable outcome using MacNab's criteria (excellent and good)/Nurick (0-2) of the last postoperative follow-up to find differences (A). Funnel plot of favorable outcome when comparing the endoscopic and microscopic tubular groups (B). Sensitivity analysis using a single-study-removal method of favorable outcome when comparing the endoscopic and microscopic tubular groups (C). Forest plots showing pooled proportion of percentage change in favorable outcome after removing studies(D). **Fig S6.** Independent samples test the blood loss between two minimal groups (A). Independent samples test the operation time between subgroups (B). Hypothesis test summary about the postoperative length of hospital stays between subgroups (C). **Table S1.** The key findings of the traditional versus endoscopic approaches in lesions.

## Data Availability

The data that support the findings of this study are available upon reasonable request from the corresponding author.
