# Supplementary material for: Efficacy of minimally invasive tubular approaches for management of the lumbar spinal synovial cysts: a meta-analysis
Source: Eur J Med Res. 2023 Nov 8;28:494. doi: 10.1186/s40001-023-01481-0 (PMC10631136; doi:10.1186/s40001-023-01481-0)
Supplement: Supplementary file 1 — Additional file 1: Fig. S1. The bar chart shows the annual distribution of published studies and the countries of origin of the studies. Fig. S2. The bar chart shows the annual distribution of published studies and the surgical methods used in the studies. Fig. S3. Graph showing LSCs locations (T12–L1 n=2; L1–2 n=18; L2–3 n=27; L3–4 n=269; L4–5 n=1129; L5–S1 n=269; S1–2 n=10). Fig S4. Forest plots showing pooled proportion of percentage change in the VAS scores after minimal resection of cysts (A). Funnel plots assessed the publication bias of the change in the percentage of VAS scores after minimal resection of cysts (B). Sensitivity analysis using a single-study-removal method (C). Forest plots showing pooled proportion of percentage change in the VAS scores after removing studies (D). Fig. S5. Forest plots showing subgroup analysis of minimal groups described the favorable outcome using MacNab's criteria (excellent and good)/Nurick (0-2) of the last postoperative follow-up to find differences (A). Funnel plot of favorable outcome when comparing the endoscopic and microscopic tubular groups (B). Sensitivity analysis using a single-study-removal method of favorable outcome when comparing the endoscopic and microscopic tubular groups (C). Forest plots showing pooled proportion of percentage change in favorable outcome after removing studies(D). Fig S6. Independent samples test the blood loss between two minimal groups (A). Independent samples test the operation time between subgroups (B). Hypothesis test summary about the postoperative length of hospital stays between subgroups (C). Table S1. The key findings of the traditional versus endoscopic approaches in lesions. [file 40001_2023_1481_MOESM1_ESM.docx]

**Additional Materials**

*for*

**Efficacy of minimally invasive tubular approaches for management of the lumbar spinal synovial cysts: A meta-analysis**

The supplementary materials include 6 supplementary figures and 1 supplementary table.


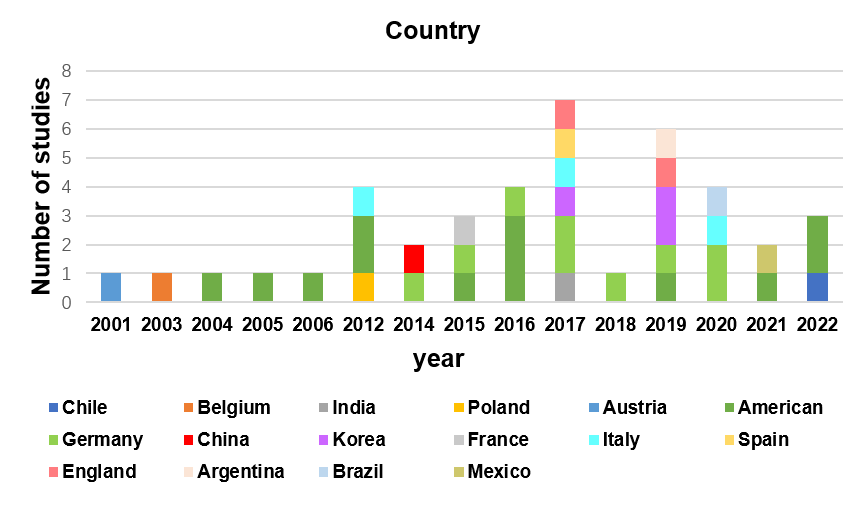


**Fig. S1** The bar chart shows the annual distribution of published studies and the countries of origin of the studies


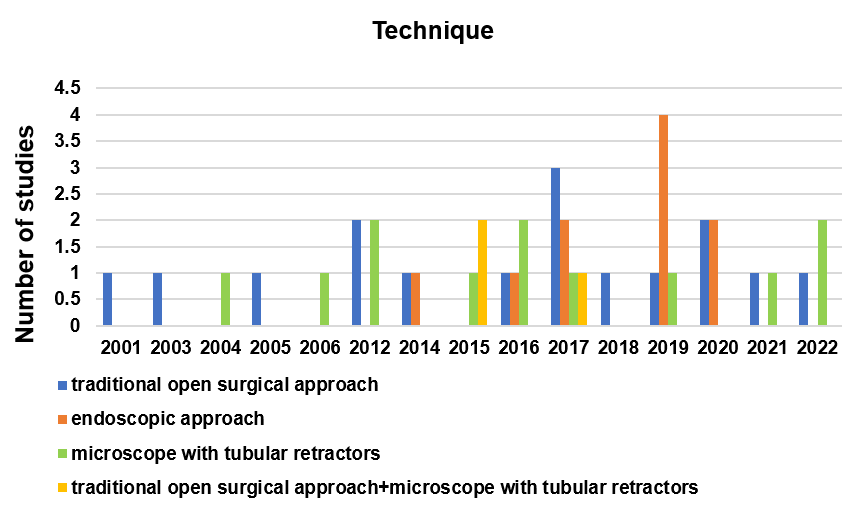


**Fig. S2** The bar chart shows the annual distribution of published studies and the surgical methods used in the studies


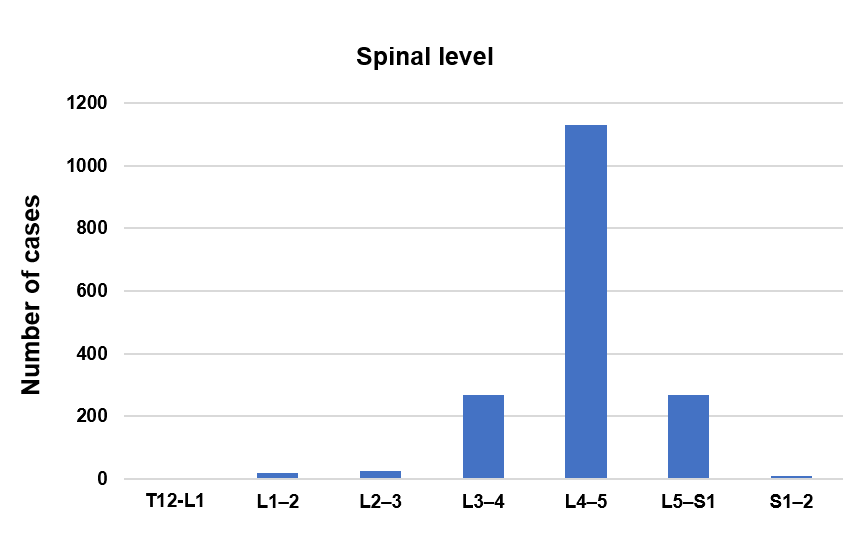


**Fig. S3** Graph showing LSCs locations (T12-L1 n=2; L1–2 n=18; L2–3 n=27; L3–4 n=269; L4–5 n=1129; L5–S1 n=269; S1–2 n=10)


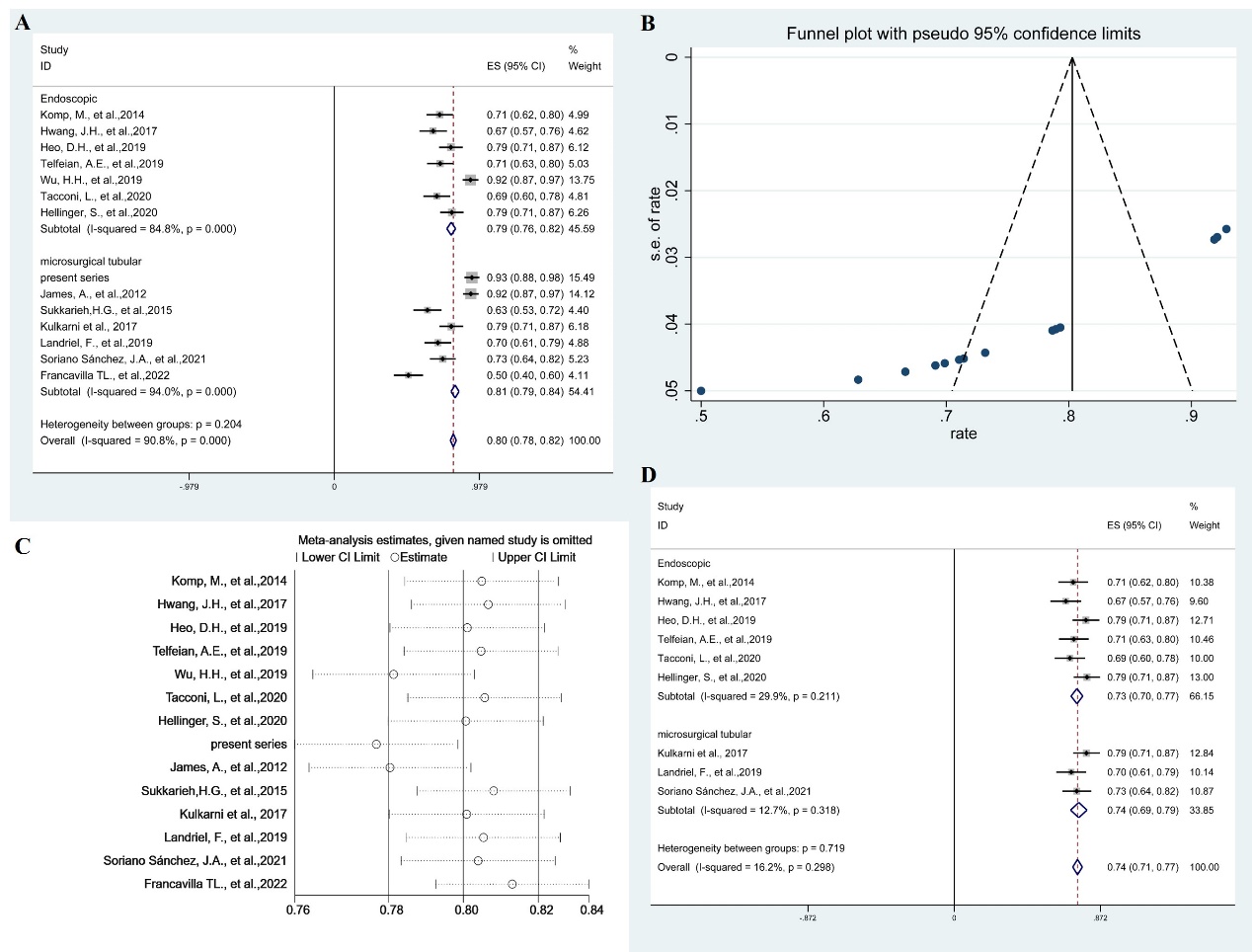


**Fig S4** Forest plots showing pooled proportion of percentage change in the VAS scores after minimal resection of cysts (A). Funnel plots assessed the publication bias of the change in the percentage of VAS scores after minimal resection of cysts (B). Sensitivity analysis using a single-study-removal method (C). Forest plots showing pooled proportion of percentage change in the VAS scores after removing studies (D).


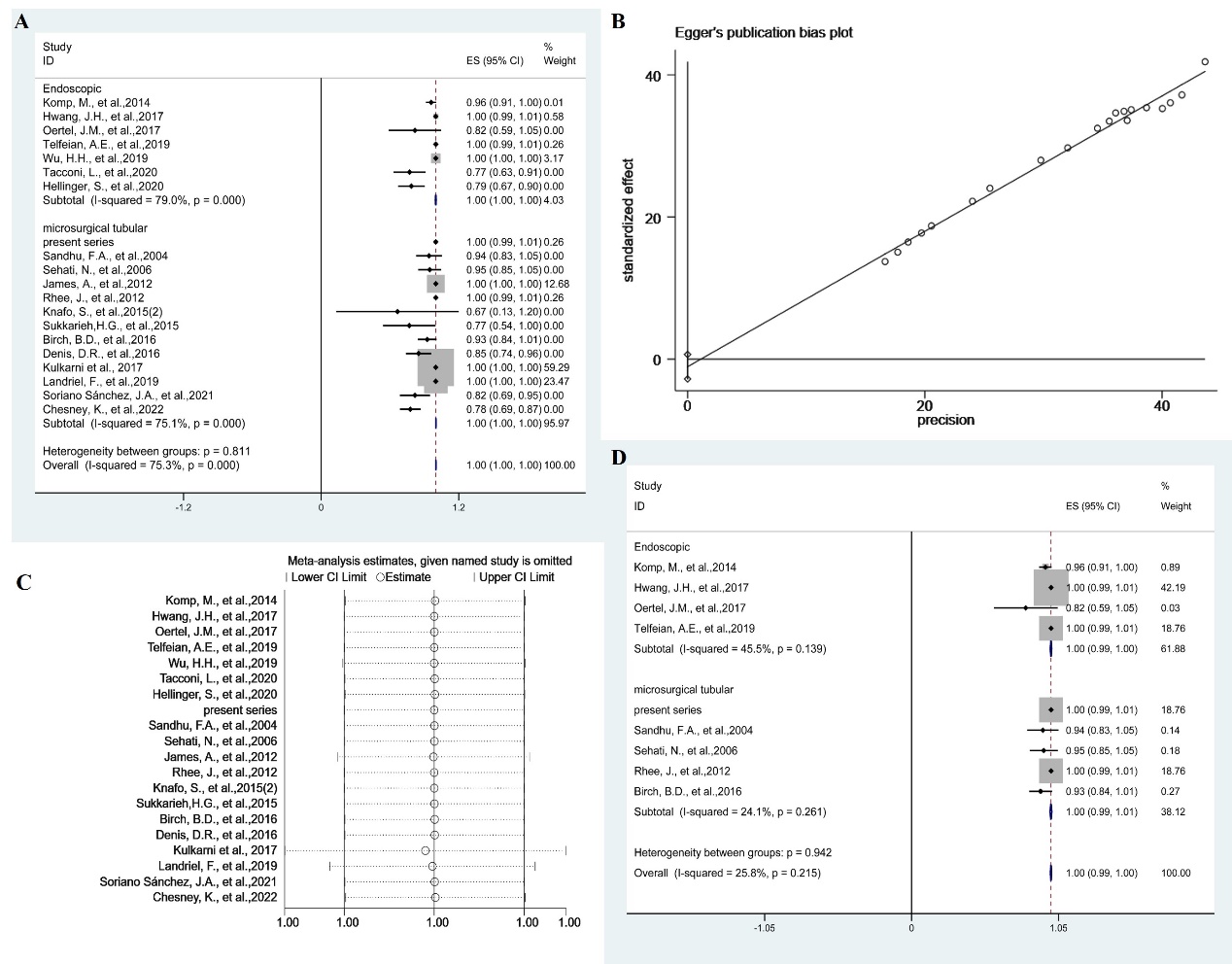


**Fig. S5** Forest plots showing subgroup analysis of minimal groups described the favorable outcome using MacNab's criteria (excellent and good)/Nurick (0-2) of the last postoperative follow-up to find differences (A). Funnel plot of favorable outcome when comparing the endoscopic and microscopic tubular groups (B). Sensitivity analysis using a single-study-removal method of favorable outcome when comparing the endoscopic and microscopic tubular groups (C). Forest plots showing pooled proportion of percentage change in favorable outcome after removing studies(D).

**
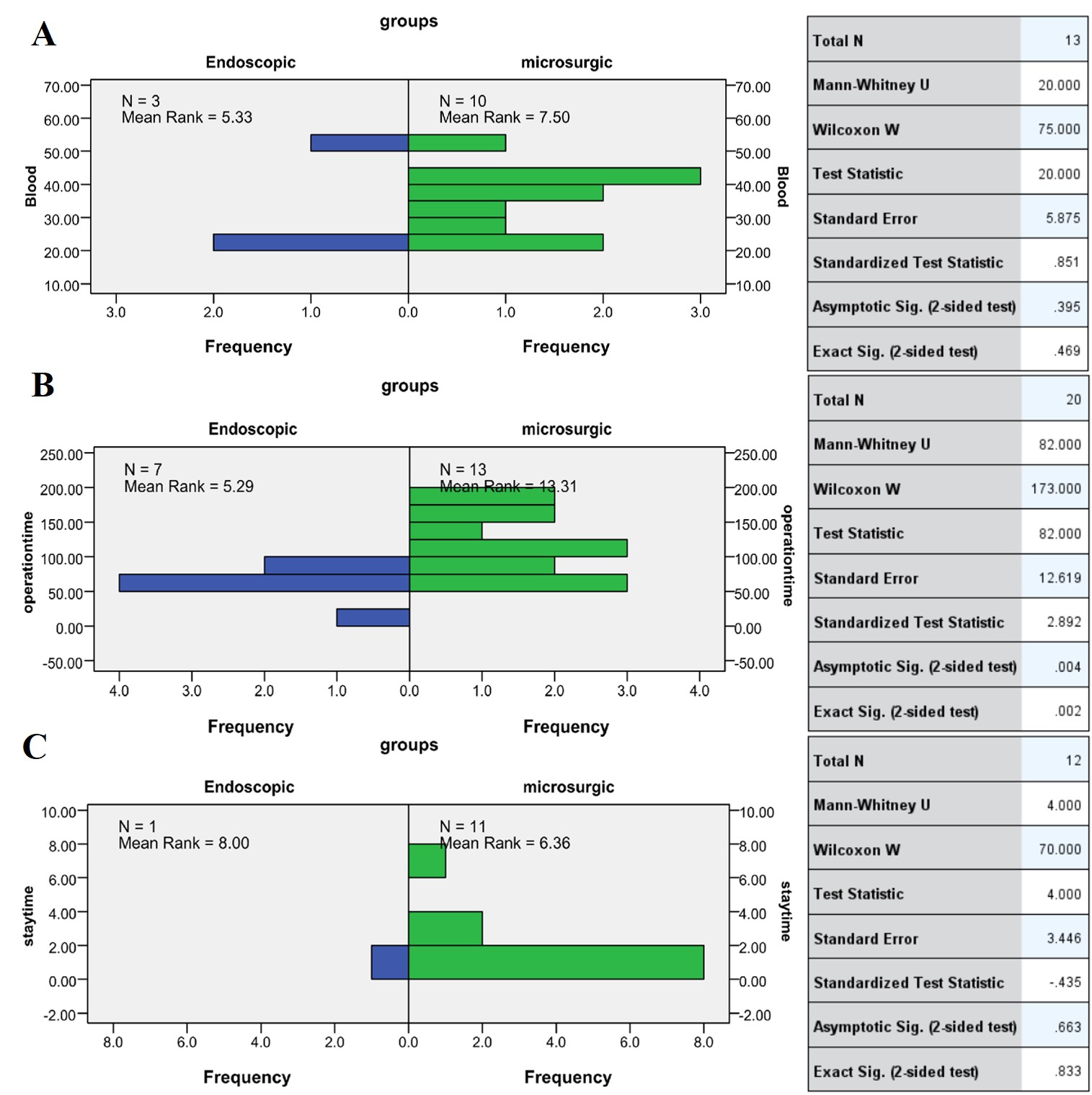
**

**Fig S6** Independent samples test the blood loss between two minimal groups (A). Independent samples test the operation time between subgroups (B). Hypothesis test summary about the postoperative length of hospital stays between subgroups (C).

**
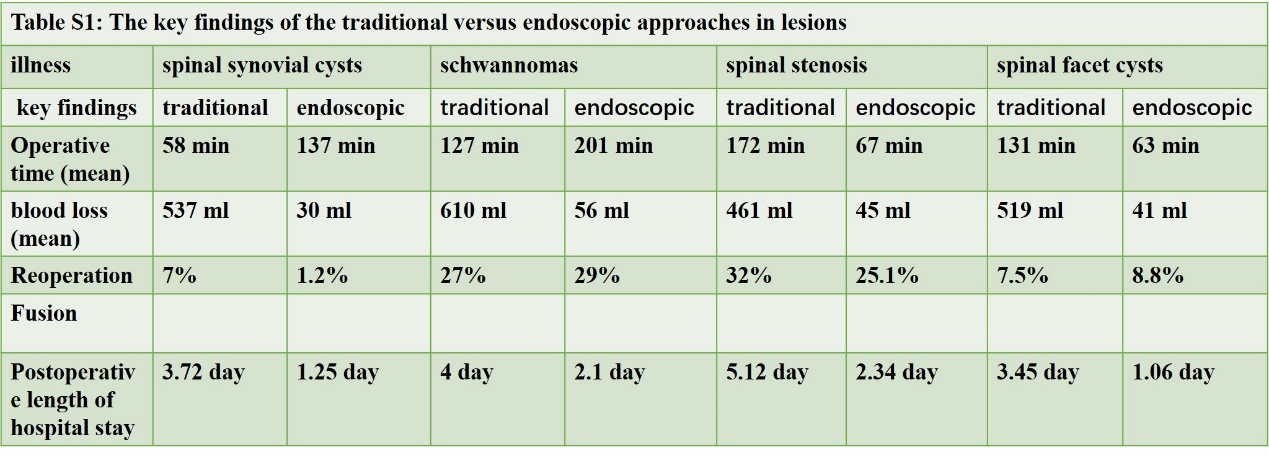
**
